# Diagnosis, management and training in perineal trauma: a UK national survey of obstetricians

**DOI:** 10.1007/s00192-023-05590-6

**Published:** 2023-07-27

**Authors:** Joanna C. Roper, Ranee Thakar, K. Joseph Hurt, Abdul H. Sultan

**Affiliations:** 1 Department of Obstetrics and Gynaecology, Croydon University Hospital, London Road, Croydon, CR7 7YE UK; 2 St George’s University of London, London, UK; 3 Divisions of Maternal Fetal Medicine and Reproductive Sciences, Department of Obstetrics and Gynecology, University of Colorado Anschutz Medical Campus, Aurora, CO USA

**Keywords:** Education, Obstetric anal sphincter injury, Obstetric perineal injury, Physicians, Training

## Abstract

**Introduction and hypothesis:**

Perineal trauma during vaginal delivery is very common. Training in diagnosis and repair of trauma, including obstetric anal sphincter injuries, varies in the UK. We aimed to investigate the current knowledge and training received by obstetric physicians.

**Methods:**

A national, validated survey was conducted online, using Qualtrics. The National Trainees Committee distributed the survey. It was also sent directly to consultants via email.

**Results:**

A total of 302 physicians completed the survey and were included in the analysis. 3.9% of participants described their training in obstetric perineal trauma as “very poor” or “poor”. 20.5% said they have not received training. 8.6% of physicians practising for more than 10 years had not had training for over 10 years. 70.5% responded “somewhat agree” or “strongly agree” when asked if they would like more training. Identification of first, second, third-, and fourth-degree tears from images and descriptions was very good (more than 80% correct for all categories). Classification of other perineal trauma was less consistent, with many incorrectly using the Sultan Classification. “Manual perineal support” and “Controlled or guided delivery” were the most frequently selected methods for the prevention of obstetric anal sphincter injury (OASI).

**Conclusions:**

Training experience for physicians in obstetric perineal trauma varies. Further improvement in training and education in perineal trauma, particularly in OASI, is needed for physicians. Perineal trauma that is not included in the Sultan Classification is often misclassified.

## Introduction

**Fig. 1:**
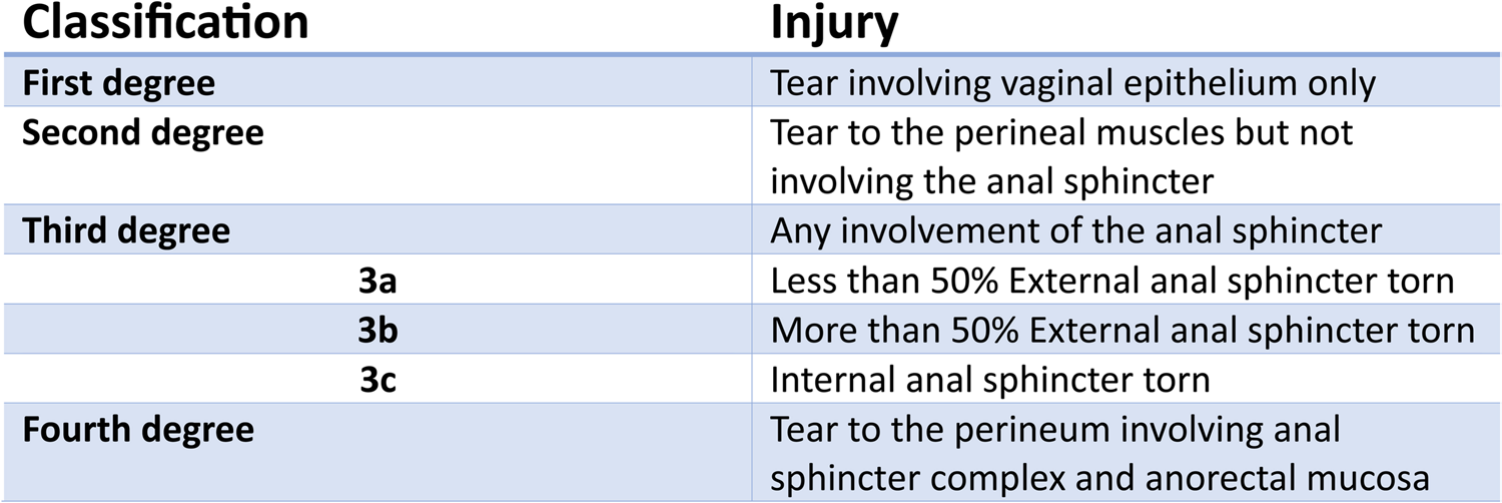
Sultan Classification for perineal tears [1]

Obstetric anal sphincter injury (OASI), also known as third- and fourth-degree tears (Fig. 1) occur in 2.9% of all vaginal deliveries (6.1% in primiparas) in the UK [2]. Short- and long-term negative effects of perineal trauma on women’s physical, emotional and social well-being are well-documented [3, 4]. In particular, although some women may experience no long-term effects from OASI, up to 61% of women suffer anal incontinence after primary repair [5].

The success of a primary repair of OASI is essential in maintaining anal continence and improving outcomes for women [6]. It is known that secondary repair achieves poor results in the long term when the severity of the incontinence is assessed [7]. In the study by Kirss et al. on factors associated with a failed primary repair, there was a statistically significant correlation between a successful repair and a more experienced surgeon [8].

Misdiagnosis and incorrect classification of perineal tears are more likely to lead to symptoms that have a significant impact on the quality of life [9, 10]. A study by Andrews et al. in 2006 showed that physicians missed 28% of OASI when patients were re-examined (after examination by the accoucheur) by an experienced research fellow [11]. Another study in 2022 of 1,056 women diagnosed with OASI found that 11.36% had a defect on endo-anal ultrasound greater than the original classification [10]. In 1995, Sultan et al. also found that a lack of knowledge of anatomy leads to under-classification and misdiagnosis of perineal trauma [12]. Wakefield et al. found that 3.6% of OASI were recorded incorrectly (in the electronic delivery summary or ICD-9 diagnostic coding) when compared with an expert review of the description of tears in the medical records [13]. Although this is a small percentage it is vital that records are accurately kept for all patients. This all implies a need for increased training.

A national practice survey in the UK in 2002 demonstrated that 33% of consultant obstetricians (672 respondents) and 22% of trainees (148 respondents) incorrectly classified a tear in the anal sphincter as a second-degree tear [14]. This was attributed to a lack of recognition, due to a lack of training. In the same survey, 64% of consultants and 64% of trainees said that there was a “lack of” or “unsatisfactory” training in the management of OASIs [14]. Since that survey, courses have been developed to train physicians and midwives in the prevention, diagnosis and management of perineal trauma including OASI [15, 16].

Training has been shown to improve the detection of OASI and the accuracy of classification [17, 18]. A quality improvement study in Palestine also showed that after education and training, there was a significant increase in the diagnosis of OASIs and this was attributed to an increase in accurate diagnosis [18]. Krissi et al. found that there was a decreased rate of OASIs following a structured hands-on workshop [17]. This was attributed to improved accuracy in the classification of trauma, and improved awareness of risk factors and prevention techniques.

Similar surveys in the USA, Australia and New Zealand have also found a need for improved training in the diagnosis and management of obstetric injuries [19–22]. In a national cohort of US obstetricians, 66% of respondents had no formal training in obstetric trauma diagnosis and classification [20], whereas 75.8% of trainees of the Royal Australian and New Zealand College of Obstetricians and Gynaecologists previously attended an OASI workshop [19]. However, 86.4% still felt that additional training would be valuable [19]. Almost all (98%) obstetrics and gynaecology resident physicians in Spain felt that there was a need for a theoretical–practical course on pelvic floor anatomy and the repair of its injuries [23].

Prevention and management of OASI are often part of the training that physicians receive. The Green-top guideline for third- and fourth-degree tears gives an evidence-based approach to the prevention, diagnosis and management of OASI for UK physicians [2]. When US physicians were asked about guidelines for the repair of obstetric lacerations, only 33.3% said that they were available in their hospital [20].

This study is aimed at identifying the current knowledge and training of obstetric physicians in obstetric perineal injuries in the UK, particularly OASI. With a rise in available training courses in obstetric perineal injuries over the last 20 years, we hypothesise that satisfaction in training and knowledge in this area has improved since Fernando’s survey in 2002 [14]. We hope that this would also mean that the detection of OASI and clinical outcomes for patients with OASI have also improved, but it is beyond the scope of this survey to determine this. By including questions relating to tears not included in the Sultan Classification we are interested to see how people categorise these traumas and if a standardised nomenclature for these is needed. We aim to raise awareness of the importance of up-to-date training in this area to ensure that OASI are not missed, leading to serious morbidity for patients.

## Materials and methods

This is a descriptive, cross-sectional study. We conducted an online survey of obstetric physicians to assess the current experience and training in perineal injuries in the UK. In collaboration with the team from the paper by Diko et al. [22], based at the University of Colorado, the survey was constructed and adapted for use in the UK.

By using an adapted version of the survey, a published survey instrument became available with the potential to compare the knowledge and practice of UK and USA physicians. The survey was designed using Qualtrics (www.qualtrics.com), which is a secure online application for building and managing surveys.

The survey was distributed to physicians via the National Trainees Committee (NTC). The NTC has many regional representatives who advertised the survey, through an open-link email invite, and encouraged participants to complete it. The survey was further advertised through Facebook© groups such as ”West Midlands Obstetrics and Gynaecology Trainees” and “Physician Mums Group UK” (PMGUK). It was also sent directly to consultants via email, using addresses available on www.nhs.net. The survey was open for 4 months.

Each participant was asked to create a unique identifier before completing the survey. This maintained confidentiality and created an opportunity to identify duplicate respondents. There was an option to enter the participant’s email address. This was held separately from the survey answers so that the participant could not be traced to their answers. The email address was used to randomly select one participant to receive a £100 voucher. This was advertised as an incentive to complete the survey.

The survey had 58 questions regarding obstetric perineal trauma diagnosis, training, and management in the survey. Included in the survey were nine images that had been drawn by a professional medical illustrator and used in a previous survey [21] of perineal tears, which the participants were asked to classify. Similarly, participants were asked to classify tears based on descriptive text.

Survey responses were included if they agreed to participate, were currently practising in the UK, have had active participation in obstetric deliveries in the past year and had worked on at least one delivery per month on average. Participants were excluded if they were not currently performing clinical work in the UK, had not been involved in obstetric activities in the past year or the survey was incomplete.

The data were securely collated in Qualtrics and analysed anonymously. Responses were collated and tables were produced to show percentages of responses to each question. For the questions involving classifying tears (images and descriptive text) “correct” responses were agreed by the writers and participants’ correct responses were recorded as a “score”. Factors such as training received and job role, associated with the score achieved were analysed. The analysis was performed in two stages. Initially, the separate, univariate, association between each factor and the outcome score was assessed. Subsequently, the joint association between the factors and the outcome was examined in a multivariate analysis. The survey project was approved by the Health Research Authority (HRA) and exempt from approval by the Institutional Review Board (IRB).

## Results

A total of 451 participants completed the survey, 302 (67%) physicians and 149 (33%) midwives. In this paper, we analysed the results of the survey completed by physicians only, as midwives have different training, do not repair OASIs and therefore will be published separately.

### Demographics

**Fig. 2:**
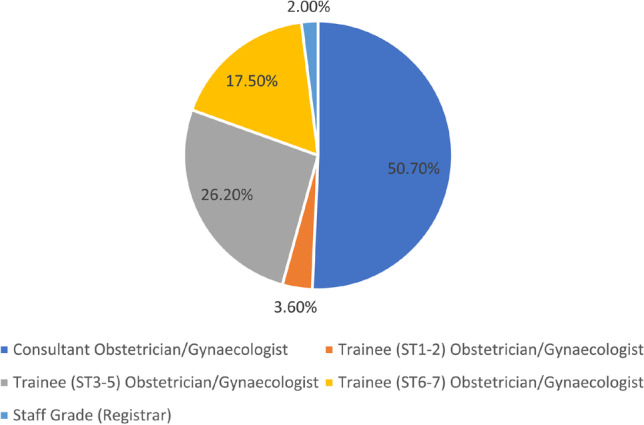
Job role of respondents

Table 1 shows the demographics of the respondent population. Consultants made up 50.7% (Fig. 2). The survey was emailed to 1,459 consultants. This gives a response rate of 10.5% for consultants. It is not possible to calculate a response rate overall because it is not possible to know how many people were reached by social media. In the UK in 2018 there were 2,600 consultants, 1,800 trainees and 1,000 speciality physicians (non-training grades) working in obstetrics and gynaecology [24]. We found that 38.7% of respondents had been working as a clinician for less than 10 years, 91.1% were working in England, and most (99.7%) respondents stated that their “main practice setting” is in a hospital delivery suite.

**Table 1:** Respondent demographics and practice characteristics

Participant characteristics (*N* = 302)	Data
Job role, *n* (%)	
Consultant obstetrician/gynaecologist	153 (50.7)
Trainee (ST1-2) obstetrician/gynaecologist	11 (3.6)
Trainee (ST3-5) obstetrician/gynaecologist	79 (26.2)
Trainee (ST6-7) obstetrician/gynaecologist	53 (17.5)
Staff grade (registrar)	6 (2.0)
Age, years, median (range)	39 (26–67)
Race, *n* (%)	
White/white British	177 (58.6)
Asian/Asian British	68 (22.5)
Any other/prefer not to answer	28 (9.3)
Black/African/Caribbean/Black British	16 (5.3)
Mixed/multiple racial or ethnic groups	13 (4.3)
Gender, *n* (%)	
Female	237 (78.5)
Male	61 (20.2)
Prefer not to say	4 (1.3)
Years in practice, including training, since university median (range)	13 (1–40)
Characteristics of current practice	
Average number of deliveries per month median (range)	10 (1–300)
Current practice setting, *n* (%)	
Hospital delivery suite	301 (99.7)
Gynaecology	1 (0.3)
Geographic region, *n* (%)	
England	275 (91.1)
Scotland	24 (7.9)
Wales	2 (0.7)
Northern Ireland	1 (0.3)

### Training

**Fig. 3:**
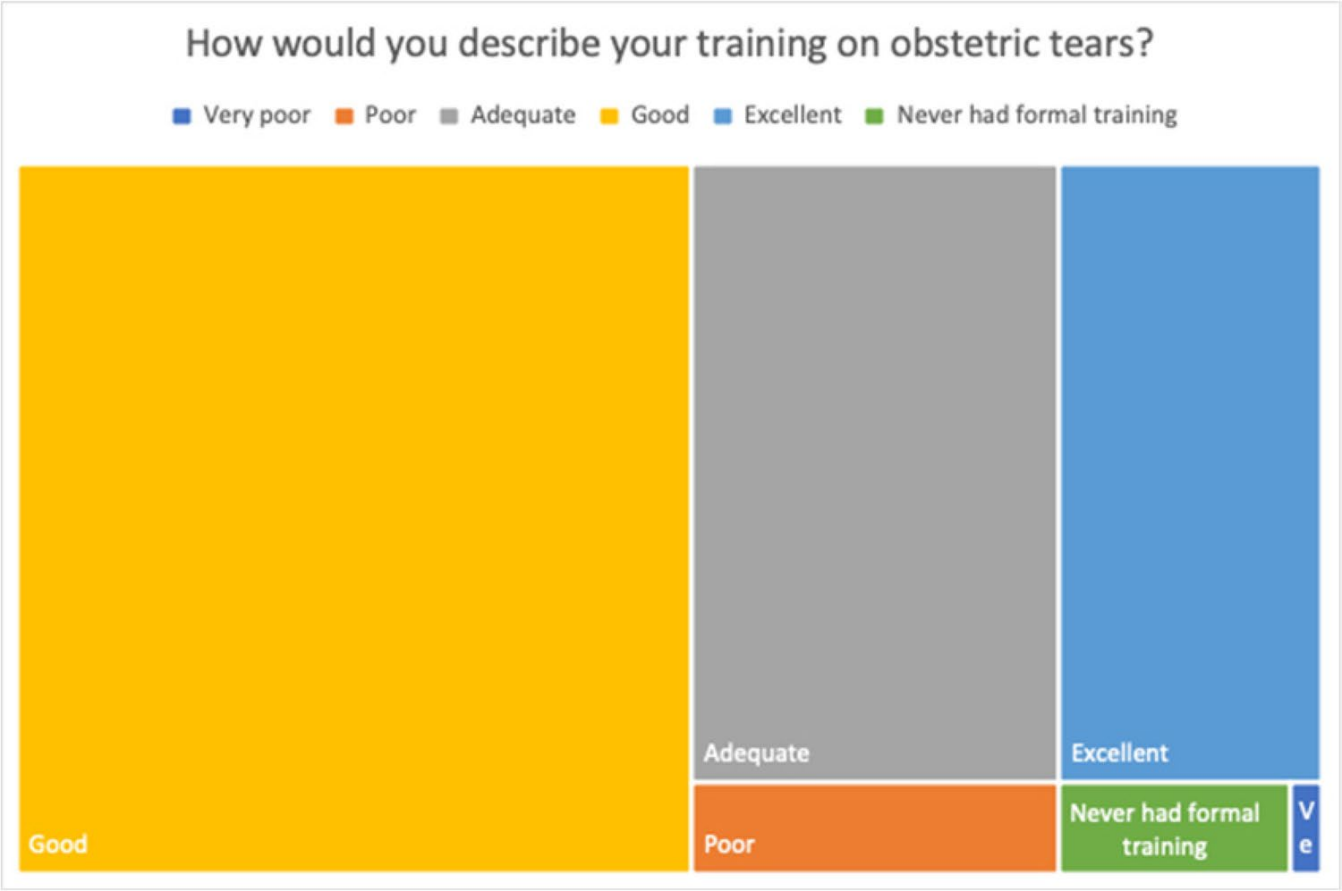
Training in obstetric tears

Most respondents (69.2%) described their training in obstetric tears as “Good” or “Excellent” (Fig. 3). Most participants (76.8%) have had training in obstetric tears and 74.6% of these were within the last 5 years (Table 2). Of those respondents practising for more than 10 years 8.6% have not had training for over 10 years. When respondents are split into consultants and juniors, 10.4% of consultants have not had training in obstetric perineal trauma for over 10 years, whereas 65.1% of junior physicians have had training in the last 5 years. Almost three-quarters (74.2%) of respondents have had training in OASI.

**Table 2:** Description of training in obstetric tears

Description	Data
How would you describe your training on obstetric tears? *n* (%)	
Very poor	1 (0.3)
Poor	11 (3.6)
Adequate	74 (24.5)
Good	156 (51.7)
Excellent	53 (17.5)
Never had formal training	7 (2.3)
Have you had CPD or training regarding obstetric tears? *n* (%)	
Yes	232 (76.8)
No	62 (20.5)
I don’t know/Unsure	8 (2.7)
How many years ago was your last CPD/training in obstetric tears? (*n* = 232), *n* (%)	
≤5	173 (74.6)
6–10	35 (15.1)
11–15	10 (4.3)
16–20	6 (2.6)
Left blank	8 (3.4)
In what learning environment was your last CPD/training on obstetric tears? (*n* = 232), *n* (%)	
Online	18 (7.8)
In person	190 (81.9)
Multi-modal (e.g. online and in person)	22 (9.5)
Other/none of the above	2 (0.9)
How long did CPD/training last in whole hours? median (range)	4 (1–25)
What topics were covered in your training? Tick all that apply, *n* (%)	
OASI	224 (74.2)
Episiotomy	149 (49.3)
Minor laceration repair	57 (18.9)
Complex laceration repair	71 (23.5)
Don’t remember/other	6 (2)
What is the main reason for no training in obstetric tears? Tick all that apply (*n* = 70), *n* (%)	
Lack of time	21 (30.0)
Lack of courses	22 (31.4)
Other (cost)	1 (1.4)
Don't know/other/none of the above	23 (32.9)
Are there any local protocols/guidelines for obstetric tears readily available to you in your practice or hospital? *n* (%)	
Yes	274 (90.7)
No	5 (1.7)
I don’t know/unsure	23 (7.6)

*CPD* continuing professional development

Of those who expressed they had had no training or were unsure if they had, 31.4% said the reason was “lack of courses available” for training. When asked if participants were interested in obtaining more education or training in obstetric perineal trauma, 70.5% responded “Somewhat agree” or “Strongly agree”.

### Prevention

Table 3 shows that the methods used by most participants to help prevent OASIs were “controlled or guided delivery” and “manual perineal support”. Almost all (99.3%) of the respondents said that they perform episiotomies, 299 (99%) said that they use a mediolateral episiotomy and 1 respondent said they use a lateral episiotomy.

**Table 3:** Methods of prevention of obstetric anal sphincter injury (OASI)

Method for prevention of OASI (tick all that apply)	Number of physicians (*N* = 302), *n* (%)
Manual perineal support	286 (94.7)
Controlled or guided delivery	280 (92.7)
Warm compresses	85 (28.1)
Perineal massage	79 (26.2)
Avoid instrumentation with forceps	53 (17.5)
Delayed pushing	39 (12.9)
Changing birthing positions	37 (12.3)
Birth preparation instruments or devices	24 (7.9)
Other (selective episiotomy)	19 (6.3)
Routine episiotomy	16 (5.3)
Avoid instrumentation with vacuum	16 (5.3)
Delivery between contractions	15 (5.0)
Physiological pushing	9 (3.0)
Dietary recommendations	7 (2.3)
Avoid episiotomy	7 (2.3)
Other (Episcissors)	6 (2.0)
None of the above	5 (1.7)
Hands-off delivery, no touching	1 (0.3)
Other	1 (0.3)
Other (avoid pool delivery)	1 (0.3)

### Diagnosis

Very few participants described their ability to identify tears as “very poor” (Table 4), but 3.6% of respondents described themselves as “poor” at identifying third-degree tears. When asked about using the sub-category system to describe third-degree tears, 100% of participants reported using this.

**Table 4:** Self-assessment of ability to identify obstetric tears

How would you describe your current ability to identify/diagnose	Very poor, *n* (%)	Poor, *n* (%)	Adequate, *n* (%)	Good, *n* (%)	Excellent, *n* (%)
First-degree tears	1 (0.3)	–	15 (5.0)	104 (34.4)	182 (60.3)
Second-degree tears	1 (0.3)	–	13 (4.3)	108 (35.8)	180 (59.6)
Third-degree tears	1 (0.3)	11 (3.6)	37 (12.3)	156 (51.7)	97 (32.1)
Fourth-degree tears	–	6 (2.0)	38 (12.6)	118 (39.1)	140 (46.4)

Tables 5 and 6 show the identification of perineal injuries from images and text descriptions. Overall, most respondents correctly identified first-, second-, third- and fourth-degree tears. Identification of other perineal injuries, e.g. labial tears, was less consistent, with many respondents incorrectly using the Sultan Classification for non-perineal injuries.

**Table 5:** Identification of obstetric lacerations from standard images

Image	First degree, *n* (%)	Second degree, *n* (%)	Third degree, *n* (%)	Fourth degree, *n* (%)	There is no tear, *n* (%)	I don't know/unsure, *n* (%)	Other/none of the above, *n* (%)
First-degree tear	**276 (91.4)**	7 (2.3)	1 (0.3)	–	5 (1.7)	3 (1.0)	10 (3.3)^a^
Second-degree tear	–	**301 (99.7)**	1 (0.3)	–	–	–	–
Third-degree tear (partially torn sphincter)	–	–	**302 (100)**	–	–	–	–
Third-degree tear (completely torn sphincter)	–	–	**277 (91.7)**	20 (6.6)	–	5 (1.7)	–
Fourth-degree tear	–	–	–	**302 (100)**	–	–	–
Cervical tear	2 (0.7)	2 (0.7)	–	–	2 (0.7)	2 (0.7)	**294 (97.4)**^**b**^
Labial laceration	68 (22.5)	21 (7.0)	1 (0.3)	–	1 (0.3)	1 (0.3)	**210 (69.5)**^**c**^
Periurethral tear	97 (32.1)	2 (0.7)	–	–	7 (2.3)	5 (1.7)	**191 (63.2)**^**d**^
Vaginal sidewall tear	127 (42.1)	64 (21.2)	–	–	2 (0.7)	1 (0.3)	**108 (35.8)**^**e**^

Bold text denotes correct answers

^a^Other included: vaginal tear (3), hymen tear (5), graze (2)

^b^Other included: cervical tear (294)

^c^Other included: labial tear (210)

^d^Other included: labial tear (32), vaginal tear (2), periurethral (150), hymen tear (2), peri-clitoral tear (3), urethral tear (2)

^e^Other included: vaginal tear (107), labial tear (1)

**Table 6:** Classification of obstetric lacerations from descriptive text

Classification of obstetric tears from descriptive text	First degree, *n* (%)	Second degree, *n* (%)	Third degree, *n* (%)	Fourth degree, *n* (%)	Other, *n* (%)	I don't know/unsure, *n* (%)
Only perineal skin torn	**242 (80.1)**	6 (2.0)	–	–	54 (17.9)	–
Only superficial posterior vaginal mucosa lacerated	**244 (80.8)**	10 (3.3)	–	–	48 (15.9)	–
Perineal tissue/muscles disrupted, EAS/IAS and rectal mucosa intact	–	**280 (92.7)**	15 (5.0)	3 (1.0)	3 (1.0)	1 (0.3)
EAS torn partially (less than 50% thickness)	–	1 (0.3)	**297 (98.3)**	2 (0.7)	2 (0.7)	–
EAS torn partially (more than 50% thickness)	–	–	**299 (99.0)**	1 (0.3)	2 (0.7)	–
EAS torn completely, IAS intact, rectal mucosa intact	1 (0.3)	–	**297 (98.3)**	2 (0.7)	2 (0.7)	–
EAS torn, IAS torn, and rectal mucosa torn	–	–	5 (1.7)	**293 (97)**	4 (1.3)	–
Vaginal sidewall laceration with intact perineum	117 (38.7)	48 (15.9)	1 (0.3)	–	**130 (43.0)**	6 (2.0)
Periurethral laceration with intact perineum	75 (24.8)	8 (2.6)	–	–	**215 (71.2)**	4 (1.3)
Periclitoral laceration with intact perineum	79 (26.2)	5 (1.7)	–	1 (0.3)	**213 (70.5)**	4 (1.3)
Vaginal sulcal laceration with intact perineum	132 (43.7)	35 (11.6)	–	–	**124 (41.1)**	11 (3.6)
Cervical laceration with intact perineum	5 (1.7)	2 (0.7)	–	–	**290 (96.0)**	5 (1.7)
Labial laceration with intact perineum	82 (27.2)	3 (1.0)	1 (0.3)	–	**214 (70.9)**	2 (0.7)
EAS intact, IAS intact, with small high vaginal and rectal mucosa tear	7 (2.3)	10 (3.3)	8 (2.6)	138 (45.7)	**132 (43.7)**	7 (2.3)

Bold text denotes correct answers

For this question we did not offer participants the option to define “Other”

*EAS* external anal sphincter, *IAS* internal anal sphincter

### Management

When asked about post-partum care and repair of OASIs, 82.8% of respondents said that they routinely perform a per rectal examination after every vaginal delivery (Table 7). When asked about routine practice regarding repair of OASI most respondents (94%) said that they perform a rectal examination before and after repair. Only 75.8% said that they would give antibiotics after repair, whereas 85.1% said that they would give antibiotics during repair.

**Table 7:** Other practices by participants regarding delivery and OASI

Description	Data
When do you typically perform a digital rectal examination after vaginal delivery? *n* (%)	
Routinely after every vaginal delivery	250 (82.8)
Only if the perineum is torn	40 (13.2)
Only if the perineum is torn deeply	9 (3)
Other (after instrumental deliveries)	2 (0.7)
Other	1 (0.3)
Which of the following do you routinely perform at the time of repairing OASI? Tick all that apply, *n* (%)	
Rectal examination after repair	284 (94)
Move to the operating room for repair	284 (94)
Rectal examination before repair	283 (93.7)
Additional anaesthesia	269 (89)
Immediate repair	267 (88.4)
Antibiotics before or during repair	257 (85)
Antibiotics after repair	229 (75.8)
Irrigation of wound	39 (12.9)
Consult another specialist or another provider	31 (10.3)
Other (laxatives)	18 (6)
Bowel preparation or enema	9 (3)
Other (physio)	4 (1.3)
Other (follow-up clinic)	3 (1)

### Factors associated with the diagnosis of obstetric trauma

In Tables 8 and 9 the only factor that was significantly associated with the score obtained was age. The results suggest that older participants might have had higher outcome scores, i.e. more correct answers (when classifying tears from images and descriptive text).

**Table 8:** Univariate associations with an overall score

Outcome	Category	*n*	Mean score ± SD	Coefficient (95% CI)	*p* value
Position	Consultants	153	80.2±12.7	0	0.25
	Trainee (ST6-7)	53	78.6±11.7	−1.6 (−5.6, 2.4)	
	Trainee (ST1-5)^b^	96	77.4±13	−2.7 (−6.0, 0.6)	
Age (continuous)^a^	–	302	–	1.8 (0.1, 3.5)	**0.04**
Age category	≤35	112	77.0±13.3	0	**0.04**
	36–50	140	79.4±12.3	2.4 (−0.8, 5.6)	
	>50	50	82.4±12.4	5.4 (1.2, 9.7)	
Gender	Female	237	78.9±12.7	0	0.88
	Male	61	78.6±12.9	−0.3 (−3.9, 3.3)	
Deliveries/month	≤10	166	79.9±12.8	0	0.18
	>10	136	77.9±12.8	−2.0 (−4.9, 0.9)	
Years in practice	≤15	181	78.2±12.9	0	0.22
	>15	121	80.1±12.6	1.8 (−1.1, 4.8)	
Self-assessment of training	Very poor–adequate^c^	93	77.0±13.9	0	0.07
	Good	156	79.3±12.3	2.3 (−1.0, 5.6)	
	Excellent	53	82.0±11.6	5.0 (0.7, 9.3)	
Training in obstetric tears	No	70	79.6±12.8	0	0.65
	Yes	232	78.8±12.8	−0.8 (−4.2, 2.7)	
Protocols available	No	28	80.6±13.0	0	0.50
	Yes	274	78.9±12.8	−1.7 (−6.7, 3.3)	
Involved in lawsuit	No	283	79.3±12.6	0	0.12
	Yes	18	74.4±14.5	−4.9 (−11.0, 1.2)	

^a^Regression coefficient presented for a 10-year increase in age

^b^Staff grades (registrars) also included in this category

^c^Combined category of: very poor, poor, adequate + no training

Bold entries denotes statistically significant results

**Table 9:** Multivariate associations with overall score

Analysis	Outcome	Category	Coefficient (95% CI)	*p* value
1	Age (continuous)^a^	–	1.9 (0.3, 3.6)	**0.02**
	Involved in lawsuit	No	0	0.07
		Yes	−5.7 (−11.8, 0.4)	
2	Age category	≤35	0	**0.02**
		36–50	2.9 (−0.3, 6.1)	
		>50	5.7 (1.5, 10.0)	
	Involved in lawsuit	No	0	0.06
		Yes	−5.9 (−12.0. 0.3)	

^a^Regression coefficient presented for a 10-year increase in age

Bold entries denotes statistically significant results

When asked if they had ever been involved in a lawsuit ensuing from an obstetric tear or complications thereof 18 (6%) respondents said “Yes”.

## Discussion

This survey provides useful information about current training in obstetric perineal trauma for physicians in the UK. The main findings from our survey are very encouraging in terms of the proportion of respondents who have received training in perineal trauma and most respondents were able to correctly classify tears, within the Sultan Classification [1, 2], from pictures and descriptions.

Most respondents (76.8%) had received training in perineal trauma. However, this means that some respondents have not had training, which could hugely impact their knowledge and clinical skills. What was reassuring about our data from the assessment of their knowledge in the identification and classification of perineal trauma was that most respondents were able to correctly classify tears within the Sultan Classification system (first- to fourth-degree tears). This implies that even though some respondents have not had formal training, their knowledge was still good. Compared with Fernando’s survey from 2002, which found that 64% of physicians reported “a lack of” or “unsatisfactory” training in the management of OASI [14], there seems to have been an increase in the percentage of physicians who have received training.

The percentage of physicians who had attended training courses in Australia and New Zealand was similar (75.8%) [19], whereas in the American survey, only 44% had had formal training in obstetric trauma classification and diagnosis [20]. This shows a huge improvement in training in obstetric tears in the last 20 years in the UK. Availability of training, particularly specialist perineal trauma courses, has improved, resulting in better satisfaction in training and likely an improved knowledge in this area.

It was interesting to see that 70.5% of respondents still said that they would like more training. Given the consequences of a missed tear, particularly an OASI [9, 10] or a buttonhole tear [25], such as incontinence, it is obviously a process that physicians are eager to carry out correctly. Some said that their lack of training was due to a “lack of available courses” indicating a need for even more training courses. It is possible that this answer was affected by COVID-19 restricting courses during the pandemic. A UK survey in 2015 on OASI management and training found that over one-third of the 104 respondents’ hospitals did not provide training in OASI [26]. The variation in training availability and the differences in subjects covered in the courses available likely means that training is inconsistent. There is a need for continuity across the training courses so that all physicians receive training in evidence-based techniques for the prevention, diagnosis and repair of perineal trauma.

It was encouraging to see that 57.3% of respondents had training in perineal trauma less than 5 years ago. This is likely because attending a training course is a mandatory part of speciality training in obstetrics and gynaecology in the UK. However, this implies a need for some physicians to attend an update or revisit the training. OASIs are not very common [2] and therefore experience can be difficult to build up. Knowledge and skills decrease over time after a training course. A study of 116 health care professionals attending a Newborn Life Support course in Greece found that theoretical knowledge was significantly reduced when assessed at 3 and 6 months [27]. They also found that some technical skills decline in performance over time after the course [27]. When asked for suggestions for improving training in OASIs, 38.6% of Australian and New Zealand physicians asked for mandatory workshops, with some suggesting that these should be yearly [19]. We propose that attending a refresher course, every 3 to 5 years, could improve knowledge and confidence in the management of perineal trauma.

Looking in more detail at the identification of obstetric lacerations from images and descriptive text for first- and second-degree tears, more respondents correctly identified the image than the description. Both were high, but perhaps this shows a little less understanding of classification, as one will expect it to be the same if participants have a full understanding of the classification descriptions. Third and fourth-degree tear identification was even better. These results indicate that most physicians are well-educated in the classification of OASI. This is likely due to the training they have received. When compared with results from the American survey, the UK physicians were slightly better at identifying and classifying perineal tears [20] (third-degree tears from description: UK 98.3%, 99% and 98.3%; American 87%, 90.2% and 92.7%). Both surveys used standardised images and text, which are easier to classify than a patient in a clinical setting, who may have bleeding and pain. But this is certainly a good indication of clinical practice and understanding.

The identification of non-perineal injuries, such as cervical tears, was not as accurate. Many respondents were inappropriately using the Sultan Classification [1, 2] for these injuries. This was also found in the American survey [20]. We suggest that a standardised nomenclature might be designed for non-perineal tears to clarify the way in which these tears should be described.

There are many indicators from our results that imply good knowledge and education in the prevention of obstetric tears in the UK. “Manual perineal support” and “controlled or guided delivery” were the highest selected answers for preventing OASI. This is recommended in the Royal College of Obstetricians and Gynaecologists (RCOG) Green-top guideline [2]. Almost all respondents said that they use mediolateral episiotomy, which is known to be safer in the reduction of OASI than midline episiotomy [28]. In the US survey, the majority (58%) were still using a midline episiotomy [20]. This is likely to be due to the implementation of the OASI care bundle, which promotes these practices [29].

A thorough digital rectal examination after every vaginal delivery means that perineal trauma is less likely to be missed [30]. Most (82.8%) participants said that they perform a per rectal examination after every vaginal delivery, which is very encouraging. However, 13.2% said they that would only perform a rectal examination if there was a visible external tear. This could mean that some tears are missed completely. A buttonhole tear can be present without any external trauma and, if missed, can have serious complications, including recto-vaginal fistula [5]. We also found that the identification of a buttonhole tear from the descriptive text was poor. Less than half (45.7%) of respondents labelled the description as a fourth-degree tear, which it is not. Given the difficulty in diagnosis and the devastating consequences of missing this type of trauma, we believe that it is essential that training in the diagnosis and effective repair of buttonhole tears is improved. This is highlighted in our case series of buttonhole tears with a proposed repair technique [25].

There are many strengths to this study. To our knowledge, it is the largest UK national survey of obstetricians on this subject since Fernando’s survey in 2002 [14]. We included only active clinicians, by asking if they had conducted deliveries in the last year, which means the results represent current practice. The survey used was validated and used in previous studies in the USA [20–22]. In the survey, knowledge of the classification of tears was assessed in two ways, through pictures and through text. This gives a deeper representation of understanding of the classification system. We also used the Checklist for Reporting Results of Internet E-Surveys tool for the survey reporting [31].

There are also some limitations. The number of responses was low; therefore, it is difficult to extrapolate the results to the whole of the UK. However, it is difficult to know the number of physicians the survey reached, as social media was used to advertise, to calculate a response rate. Compared with other similar surveys, the number of included responses is much higher [19, 20]. It has been noted that physician web-based surveys often have a low response [32]. The spread of responses and diversity across the UK was limited. This is likely because the survey was distributed from England. The survey data capture a snapshot of information at that time and may not reflect an ever-changing educational environment. Self-reporting practices, such as post-operative care, rely on honest answers from respondents.

The survey used a monetary incentive to encourage respondents to participate. This could have skewed who decided to respond. It is also possible that physicians who have a particular interest in perineal tears or OASI, and are therefore more educated in this area, were more likely to complete the survey. This could have skewed the results. Finally, given that diagnosis of perineal injuries in the clinical environment can be more difficult when the patient is in pain, has bleeding or if the lighting is poor, this may reduce the ability of physicians to classify tears compared with the standardised images and descriptions from the survey.

## Conclusion

Knowledge of perineal trauma seems to have improved in the last 20 years. We feel that there is still further improvement needed in training and education, particularly in OASIs. A standardised nomenclature for non-perineal injuries could help to improve diagnosis and classification. We also suggest that there is a need for improved training in the diagnosis of buttonhole tears, including digital rectal examination after every vaginal delivery. Finally, more research is needed on how often a refresher course is required to ensure that knowledge and skills are maintained without any detriment to patient care.
